# Second-line virologic failure and elevated bilirubin as a potential surrogate marker of ART adherence among people living with HIV in Eastern Uganda

**DOI:** 10.1186/s12981-026-00861-w

**Published:** 2026-02-19

**Authors:** Simiyu Melap Lynnet, Jacob Stanley Iramiot, Rebecca Nekaka, Patrick Okware, Mary Abwola Olwedo, Joshua Epuitai, Paul Oboth, Herbert Itabangi, Lydia V. N. Ssenyonga, Julius Nteziyaremye, David Okia

**Affiliations:** 1 Department of Nursing, Faculty of Health Sciences, Busitema University, Mbale, Uganda; 2 Department of Microbiology and Immunology, Faculty of Health Sciences, Busitema University, P.O. Box 1460, Mbale, Uganda; 3 Department of Community and Public Health, Faculty of Health Sciences, Busitema University, Mbale, Uganda; 4 Department of Paediatrics and Child Health, Soroti University, Soroti, Uganda; 5 Department of Obstetrics and Gynaecology, Faculty of Health, Busitema University, Tororo, Uganda

**Keywords:** Second-line virological failure, Elevated bilirubin, And adherence

## Abstract

Second-line antiretroviral therapy (ART) failure remains a challenge in HIV Programs. We conducted a cross-sectional study among people living with HIV on second-line ART in Eastern Uganda to determine the prevalence and associated factors of virological failure and to assess elevated serum bilirubin as a surrogate marker of adherence. The prevalence of virological failure was 7.5%. Elevated bilirubin showed poor sensitivity and specificity for predicting adherence or virological failure. The findings highlight the need for routine viral load monitoring, as bilirubin is not a reliable surrogate marker of treatment adherence or virological failure.

## Background

The rapid scale-up of the use of antiretroviral therapy has led to a global decline in the prevalence of HIV and new infections in the past ten years [1, 2]. Despite these improved outcomes in many people living with HIV (PLWHIV) on ART, some eventually fail on first-line and second-line treatment, resulting in increased morbidity and mortality [3].

Elevated bilirubin has been proposed as a proxy for adherence, which in turn influences virological outcome than pill count adherence in clients receiving atazanavir/ritonavir-based second-line antiretroviral therapy [4]. The use of elevated serum bilirubin as a surrogate marker to monitor adherence and virological treatment failure of the ATV/r-based second-line regimen is not a current practice in Uganda. Additionally, there is a paucity of data concerning second-line ART virological failure in Uganda. Addressing concerns of treatment failure is key to achieving the 95-95-95 targets by 2030, particularly viral load suppression. If treatment failure increases unchecked, viral load suppression may not be reached due to limited therapeutic options and the higher costs of expensive second and third-line treatments [5]. With the US funding cuts On HIV programs, alternative marker of adherence such as bilirubin could be viable [6].This study sought to determine the prevalence and factors associated with second-line treatment virological failure and evaluate elevated serum bilirubin as a potential surrogate marker of adherence among people living with HIV in eastern Uganda.

## Materials and methods

### Study design and setting

A cross-sectional study was carried out from 20/10/2022 to 12/03/2023. The study was conducted in the ART clinics of Tororo General Hospital and Mbale Regional Referral Hospital (MRRH). Tororo General Hospital HIV has a total of 2335 clients on ART therapy of which 162 clients were on second-line regimen. The second line regimen that is used in the facility is ATV/r, LPV/r-based regimen, and dolutegravir (DTG). Similarly, MRRH runs an ART clinic daily with a total number of clients on ART 5420 of which 522 (%) are on ATZ/r-based regimen and LPV/r-based regimen-based second-line therapy.

### Study population

PLWHIV on a second-line regimen for more than six months were eligible for the study as per the Uganda Ministry of Health Clinical guidelines, while clients who declined consent were not included. The 294 study participants were selected using a sequential sampling method.

### Data collection methods

Data was collected by reviewing the antiretroviral treatment (ART) records, interviewing participants, and performing laboratory tests for total bilirubin. Liver function tests, especially bilirubin levels, were performed to assess adherence. Standard operating procedures were followed to obtain and process the blood samples.

### Data analysis and presentation

STATA (version 15.0) was used for analysis. Descriptive statistics were used to summarize the study population characteristics and the overall prevalence of second-line virological failure. Continuous variables were summarized using mean and standard deviation (SD), while categorical variables were summarized using frequency and percentages. We used bivariate analysis to identify factors associated with virological failure. Statistically significant variables were then subjected to multivariate analysis to determine the predictors of virological failure. We then used a multivariate logistic regression model fitted through the backward selection of variables to assess the adjusted effect of predictor variables on the primary outcome. Second-line antiretroviral therapy (ART) virological failure is defined as PLWHIV who are on a second-line regimen and have a high viral load level (> 1000 copies/mL) in two consecutive measurements after 6 months of treatment and on repeat tests after 3 months [7]. Sensitivity and specificity of bilirubin was computed using the two-by-two tables.

## Results

### Characteristics of the participants

Overall, 294 participants were enrolled in the study, of which 271 (92.2%) were aged 20 years and above with a mean age of 45 years (SD 13.9 years). The majority, 116 (39.5%), had been on a second-line ART regimen for about 24–36 months with a mean duration on ART of 38.5 months (SD 16.0 months), of which 44 (15.0%) had elevated bilirubin levels, and 22 (7.5%) had second-line virological failure. The prevalence of virological failure was 7.5% (Table 1).

**Table 1:** Bivariate analysis of factors associated with second-line virological failure

Variable	Suppressed n (%), *N* = 272	Persistent non-suppression n (%), *N* = 22	COR (95% CI)	*p*-value
**Age (completed years)**				
< 10 10–19 ≥ 20	3 (1.1) 14 (5.2) 255 (93.8)	2 (9.1) 4 (18.2) 16 (72.7)	Ref 0.4(0.035–3.999) 0.1 (0.016–0.659)	0.417 0.016
**Drug frequency**				
Once Twice	269 (98.9) 3 (1.1)	17 (77.3) 5 (22.7)	Ref 26.4 (5.809 −119.735)	0.001
**Alcohol consumption**				
Yes	48 (17.7) 224 (82.4)	8 (36.4) 14 (63.6)	8.2 (3.245–20.552) Ref	0.001
**Disclosure**				
No	30 (11.0) 242 (88.8)	6 (27.3) 16 (72.7)	0.3 (0.120–0.910) Ref	0.032
**Backbone**				
ABC AZT TDF	13 (4.8) 23 (8.5) 236 (86.8)	6 (27.3) 3 (13.6) 13 (59.1)	Ref 0.3 (0.060–1.323) 0.1 (0.039–0.365)	0.109 0.001
**Protease/integrase**				
ATV/r LPV/r	66 (24.3) 1 (0.4)	11 (50.0) 2 (9.1)	Ref. 12.2 (1.016 −146.005)	0.05
DTG	205 (75.4)	9 (40.8)	0.1 (0.107–0.676)	0.01
**Duration on 2nd line ART (months)**				
12–24 24<−36 36<−48 > 48	34 (12.5) 105 (38.6) 107 (39.3) 26 (9.6)	0 (0.0) 11 (50.0) 3 (13.6) 8 (36.4)	1 empty 0.4 (0.131–0.975) 0.1 (0.023–0.377) Omitted	**0.044** **0.001**
**Most recent CD4 count (cells/mm3)**				
< 200 > 200	39 (14.3) 233 (85.7)	10 (45.5) 12 (54.6)	0.2 (0.071–0.441) **Ref**	0.001
**Adherence to ART**				
Good Poor	272 (100) 0 (0.0)	0 (0.0) 22 (100.0)	Perfectly predict the outcome	
**Most recent WHO-clinical stage**				
One Two Three Four	152 (55.9) 81 (29.8) 34 (12.5) 5 (1.8)	4 (18.2) 8(36.4) 9 (40.9) 1 (4.6)	**Ref** 3.8 (1.097–12.842) 10.1 (2.925–34.589) 7.6 (0.714–80.932)	**0.035** **0.001** 0.093
**Bilirubin level (mg/dl)**				
Elevated Normal	38 (14.0) 234 (86.0)	6 (27.3) 16 (72.7)	0.4 (0.159–1.176) **Ref**	0.101

Empty refers to those categories falling on the side of the dependent outcome, ref-reference, COR- crude odds ratio, ABC- abacavir, TDF-tenofovir, DTG- dolutegravir, GOVT- government employee, NGO- non-government organization employee, ART-Antiretroviral therapy

The age of the participant, drug frequency, alcohol consumption, duration on second line ART, most recent CD4 count, adherence to Art and the most recent WHO-clinical stage were associated with virological failure.

**Table 2:** Multivariate analysis of factors associated with second-line virological failure

Variable	COR (95% CI)	*p*-value	AOR (95% CI)	*P*-value
**Age (years)**				
< 10 10–19 ≥ 20	Ref 0.4 (0.035–3.999) 0.1 (0.016–0.659)	0.417 **0.016**	0.7 (0.013–40.476) 1.0 (0.023–41.343)	0.881 0.985
**Drug frequency**				
Once Twice	Ref 26.4 (5.809–119.735.809.735)	0.001	Ref 2.4 (0.137–41.659)	0.550
**Alcohol consumption**				
Yes	8.2 (3.245–20.552) Ref	0.001	5.2 (1.224–21.764) Ref	0.025
**Disclosure**				
No	0.3 (0.120–0.910) Ref	0.032	0.5 (0.054–5.319) Ref	0.593
**Backbone**				
ABC AZT TDF	Ref 0.3 (0.060–1.323) 0.1 (0.039–0.365)	0.109 0.001	0.3 (0.019–3.757) 0.1 (0.014–0.807)	0.325 **0.030**
**Protease/integrase**				
ATV/r LPV/r DTG	Ref 12.2(1.016–146.005.016.005) 0.1 (0.107–0.676)	**0.05** **0.01**	5.5 (0.040–63.938.040.938) 0.7 (0.138–4.547)	0.498 0.793
**Duration on 2nd line ART (months)**				
12–24 24<−36 36<−48 > 48	1 empty 0.4 (0.131–0.975) 0.1 (0.023–0.377) Omitted	**0.044** **0.001**	1 empty 0.5 (0.097–2.570) 0.08 (0.012–0.501) Omitted	0.406 **0.007**
**Most recent CD4 count (cells/mm3)**				
< 200 > 200	0.2 (0.071–0.441) **Ref**	0.001	0.07 (0.019–0.253) Ref	0.000
**Baseline WHO-clinical stage**				
One Two Three Four	**Ref** 3.8 (1.097–12.842) 10.1 (2.925–34.589) 7.6 (0.714–80.932	**0.035** **0.001** 0.093	Ref 1.9 (0.403–8.756) 2.5 (0.438–4.802) 1.5 (0.080–6.495)	0.423 0.298 0.798

The variable was considered significant when its p-value is less than 0.05 at a 95% confidence interval

## Discussion

### Prevalence of virological failure

The prevalence of second-line virological failure was found to be 7.5% using the WHO definition of virological failure of > 1000 viral copies/ml after six or more treatment cycles. This was higher compared to that found by a similar study done in Malawi [4] and much lower than reported in the earlier systematic meta-analysis about treatment outcomes of clients on second-line ART treatment in resource-limited countries [8]. The higher prevalence of virological failure in our context could be because a large proportion consumed alcohol and had a CD4 count < 200 cells/mm^3^. Despite the lower prevalence values illustrated by this finding, the second-line virological failure is of health and financial importance [9], and this undermines the efforts to achieve the 95-95-95 strategy by 2030.

### Factors associated with increased risk of developing second-line virological failure

This study found two factors posing an increased risk of developing virological failure: taking alcohol and having a the most recent CD4 count < 200 cells/mm^3^ (Table 2).

CD4 count cells have long been known to be determinants of the body’s immunity, thus enabling the body to clear viruses; however, the findings of our study have shown that having the most recent CD4 count of < 200 cells/mm^3^ increased the risk of second-line virological failure. This contradicts several studies done elsewhere [3, 10, 11].

Taking alcohol has been found in previously conducted studies to be a risk factor for virological failure [12]. This might be related to increased factors that were not within the scope of this study, like the risk of depressive symptoms, hepatitis C co-infection, and alcohol-associated low CD4 count, as was revealed in an earlier study in 2007 [13].

### Bilirubin as a surrogate marker of adherence to the ATV/r second-line ART regimen

Adherence has long been known as a determinant of virological suppression; however, the finding of this study reveals no difference in the adherence levels between virologically failing and virologically suppressing HIV-infected clients. This leaves us with two explanations: either the method of assessing adherence in place is ineffective or the emergence of resistance.

Objective biologic measures of ART adherence are more reliable in predicting poor adherence and VF than pill count or the client’s self-report, which often overestimate adherence levels. The study done earlier in Malawi revealed that normal bilirubin values in the setting of ATV/r-based therapy better predicted treatment failure than pill count. However, the findings of this study have shown that the sensitivity was found to be at 50%, with a low specificity of 31.8%. More studies are needed to support the use of bilirubin as a surrogate marker of adherence to ART.

## Conclusions

The prevalence of virological failure among people living with HIV on second-line ART regimens in two ART clinics was high. Alcohol consumption and a baseline CD4 count were independent factors associated with second-line virological failure among people living with HIV at clinics in eastern Uganda. Elevated bilirubin is not a surrogate marker for virological failure in people living with HIV on ATV/r based second-line ART regimen.

### Recommendations

We recommend continued adherence support to clients after switching to second-line treatment and continuous viral load monitoring in line with the national clinical guidelines.

A qualitative inquiry into the barriers and facilitators to understand why some PLWHIV on second-line treatment remain non adherent to contextualize interventions. Community sensitization regarding the dangers of alcohol consumption needs to be implemented, focusing more on HIV-infected populations.

Health workers need to actively identify potential advanced HIV disease and the common markers of treatment failure.

### Limitations

Participants on TB treatment, known NCDs or on other medications that may elevate bilirubin levels were not excluded from this study.

## Data Availability

The datasets generated and/or analyzed during the current study are available in the OSF repository [osf.io/mqf3b](https:/osf.io/mqf3b). Also, the corresponding author can provide the data if required.

## Abbreviations

HIV: Human immunodeficiency virus
ARV: Anti-retroviral
CD4: Cluster of differentiation
WHO: World Health Organization
ART: Anti-retroviral therapy
ATV/r: Atazanavir/ritonavir
UGT: Uridine glucuronide transferase
LPV/r: Lopinavir/ritonavir
MRRH-REC: Mbale regional referral hospital research Ethics Committee
NGO: Non-Governmental Organization
DTG: Dolutegravir
MRRH: Mbale region referral hospital
